# Gestational Polyphenol Levels and Risk of Atopic and Respiratory Outcomes in Early‐Life: Insights From the LiNA Study

**DOI:** 10.1111/all.70328

**Published:** 2026-04-03

**Authors:** Sergio Gómez‐Olarte, Carolin Huber, Stefan Röder, Ulrich Sack, Michael Borte, Martin Krauss, Werner Brack, Ana C. Zenclussen, Gunda Herberth

**Affiliations:** ^1^ Department of Environmental Immunology Helmholtz Centre for Environmental Research–UFZ Leipzig Germany; ^2^ Department of Exposure Science Helmholtz Centre for Environmental Research–UFZ Leipzig Germany; ^3^ Department of Environmental Chemistry Eawag–Swiss Federal Institute of Aquatic Science and Technology Duebendorf Switzerland; ^4^ Institute of Clinical Immunology, Faculty of Medicine Leipzig University Leipzig Germany; ^5^ Children's Hospital, Municipal Hospital “St. Georg” Academic Teaching Hospital of Leipzig University Leipzig Germany; ^6^ Department of Evolutionary Ecology & Environmental Toxicology, Faculty of Biological Sciences Goethe University Frankfurt Frankfurt am Main Germany; ^7^ German Center for Child and Adolescent Health (DZKJ) Partner Site Leipzig/Dresden Germany; ^8^ Perinatal Immunology, Saxonian Incubator for Clinical Translation (SIKT), Medical Faculty Leipzig University Leipzig Germany

To the Editor,

Maternal diet during pregnancy may potentially influence immune development and subsequent atopic risk in children [1]. Dietary antioxidant and anti‐inflammatory compounds, such as polyphenols, have been associated with a reduced risk of allergies and respiratory diseases [2]. However, most available evidence on their health effects comes from self‐reported food‐frequency questionnaires, which are intrinsically susceptible to recall and reporting biases [3]. Thus, there is a need for objective biomarkers of polyphenol internal exposure during gestation. Here, we investigated the link between maternal polyphenol markers in late pregnancy, individually and as mixtures, with the lifetime prevalence of atopic dermatitis (AD), food sensitization, wheezing, and bronchitis in 3‐year‐olds (Figure 1A and Figure S1), and assessed whether the children's type 2 blood cytokines (Table S1) may mediate their association with AD.

**FIGURE 1:**
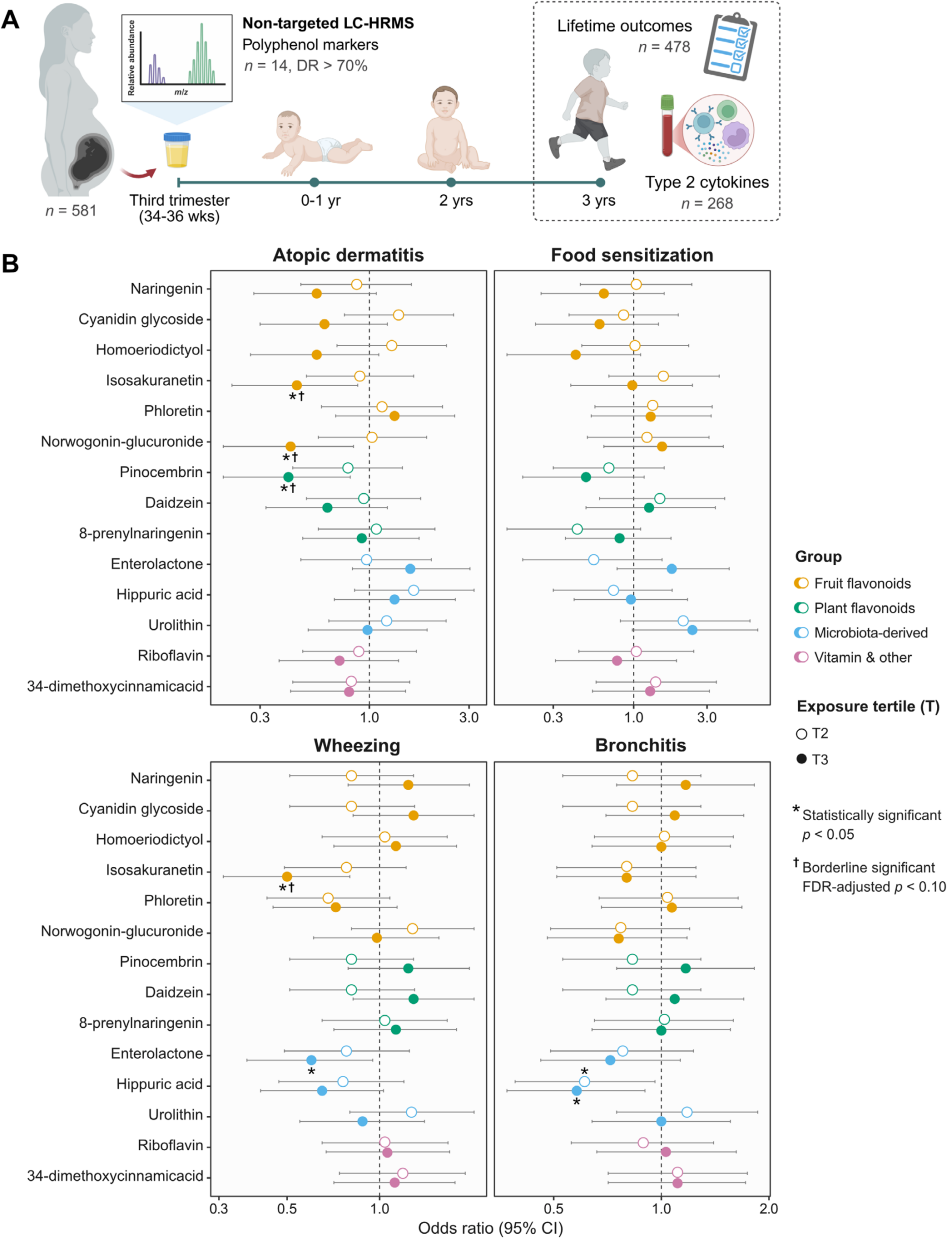
(A) Diagram that displays the study design, which included 581 mother–child pairs from the prospective birth cohort LiNA: Lifestyle and environmental factors and their influence on the Newborn Allergy risk. Polyphenol markers were measured during pregnancy by liquid chromatography/high‐resolution mass spectrometry (LC‐HRMS) and prospectively associated with childhood outcomes at age 3. (B) Forest plot of estimates linking polyphenol markers (categorized into tertiles, with T1 as reference) in pregnancy with AD, wheezing, and bronchitis (*n* = 478), and food sensitization defined by fx5‐IgE (*n* = 268) in 3‐year‐old children. All multivariable logistic regression models were adjusted for smoking/ETS exposure during pregnancy, breastfeeding up to 6 months, cat keeping, parental atopy history, parental education level, and child sex. The asterisks (*) indicate statistically significant associations (*p* < 0.05), while the daggers (†) denote those reaching borderline significance (*p* < 0.10) after adjustment for multiple testing using the Benjamini‐Hochberg false discovery rate (FDR) procedure.

Our study included 581 mother–child pairs from the prospective birth‐cohort LiNA (*n* = 622 at baseline) [4] with semiquantitative LC‐HRMS‐based measurements of 46 food markers in gestational urine samples [5] (Table S2). To capture consistent patterns of internal exposure and reduce sparse‐data bias, only polyphenol markers with a detection rate > 70% (*n* = 14) were selected for analysis (Table S3). Some polyphenols were strongly positively correlated, for example, isosakuranetin and homoeriodictyol (*r* = 0.97) or negatively correlated, such as naringenin and enterolactone (*r* = −0.83), which may reflect shared dietary sources or metabolic pathways (Figure S2B). All statistical models were fitted using the annotated LC‐HRMS signal intensities categorized into tertiles (Ts) and adjusted for environmental and lifestyle variables to control for potential confounding (details in Supporting Information).

In the 3‐year follow‐up subcohort (*n* = 478, Figure 1B), adjusted logistic regression models showed that children whose mothers ranked the upper tertile (T3) of the flavonoids isosakuranetin (aOR = 0.45, 95% CI: 0.22–0.88), norwogonin‐glucuronide (aOR = 0.42, 95% CI: 0.20–0.84), and pinocembrin (aOR = 0.41, 95% CI: 0.20–0.81) had lower odds of AD compared with those in the lowest tertile (T1, used as reference). The upper tertiles of enterolactone and hippuric acid, two microbiota‐derived compounds, were also associated with reduced odds of wheezing (aOR = 0.50, 95% CI: 0.31–0.80) and bronchitis (aOR = 0.60, 95% CI: 0.37–0.95) (Figure 1B), respectively. Next, we examined whether mixtures of major polyphenol groups detected in pregnancy may be associated with health outcomes at age 3. In the quantile g‐computation analyses, a one‐tertile increase in the mixture of flavonoids was associated with a reduced odds of AD (aOR = 0.50, 95% CI: 0.27–0.92; *p* = 0.027), with pinocembrin and norwogonin‐glucuronide having the largest weights (33% and 23%) in the overall estimate (Figure 2A). Among microbiota‐derived compounds, hippuric acid and enterolactone contributed equally (weights: 44%) to the mixture model linked with a lower wheezing risk (aOR = 0.68, 95% CI: 0.48–0.96; *p* = 0.029) (Figure S3).

**FIGURE 2:**
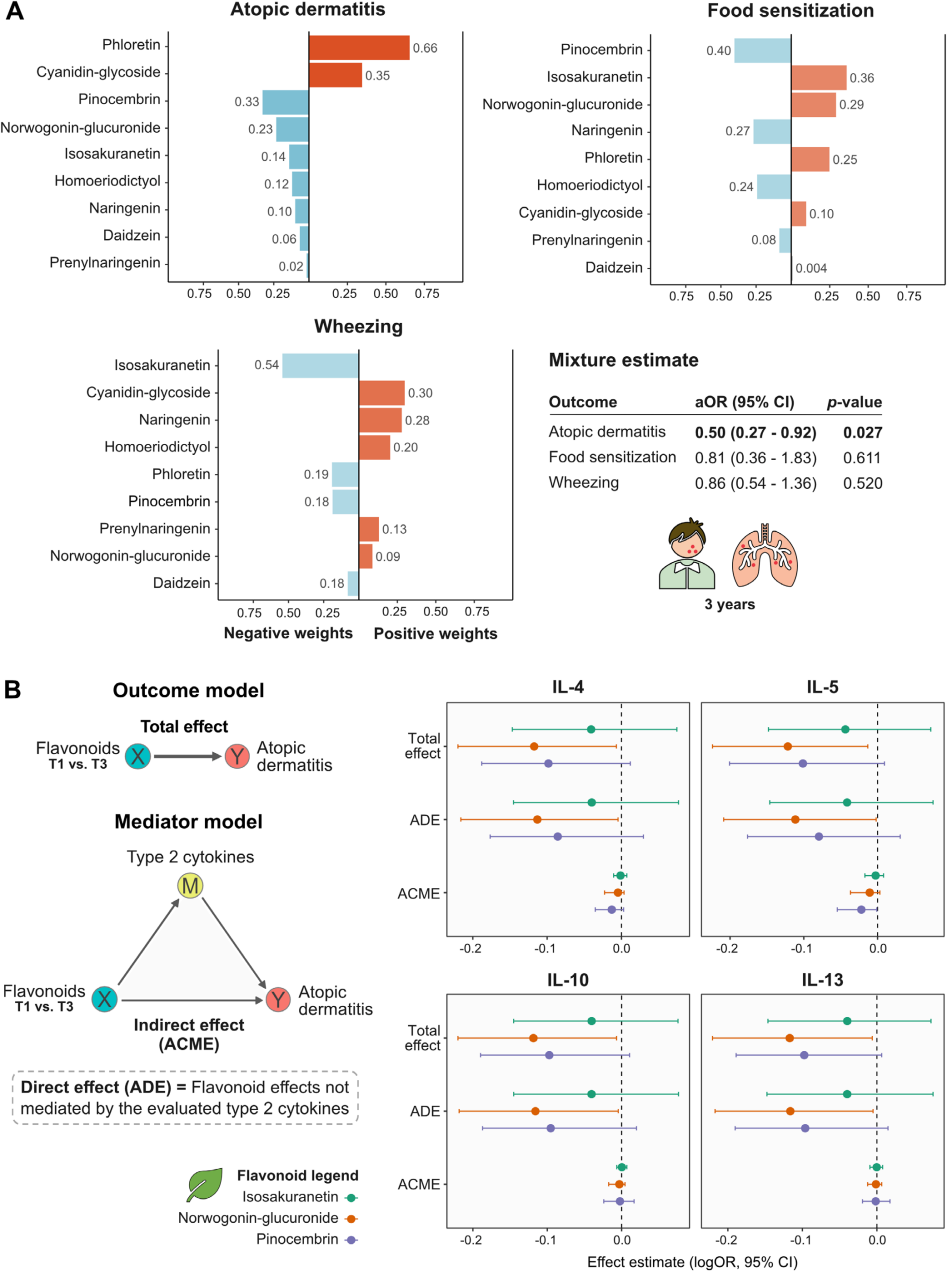
(A) Quantile g‐computation analysis on the association between a mixture of 9 flavonoids measured in pregnancy and health outcomes in children aged 3. The three models were fitted using polyphenol markers quantized into tertiles (q = 3) and adjusted for smoking/ETS exposure during pregnancy, breastfeeding up to 6 months, cat keeping, parental atopy history, parental education level, and child sex. (B) Flow chart and estimate plots of the mediation analyses that assess the indirect association of children's type 2 blood cytokines (IL‐4, IL‐5, IL‐10, and IL‐13) on the inverse relationship between three flavonoids and AD among mother–child pairs at year 3 (T3 vs. T1, *n* = 178).

Lastly, we performed mediation analyses to assess whether the children's type 2 cytokine profile (*n* = 268) was consistent with the observed inverse relationship between isosakuranetin, norwogonin‐glucuronide, and pinocembrin and AD. The indirect association via IL‐5 was statistically significant (−0.022, 95% CI: −0.055, −0.001, *p* = 0.042), whereas the direct association in the outcome model reached only borderline significance (−0.101, 95% CI: −0.201, 0.009, *p* = 0.079) (Figure 2B). This trend aligns with in vitro and in vivo studies on certain phytochemicals' capacity to modulate Th2‐signaling, involving IL‐5 and NF‐κB pathways [6].

Altogether, we identified five polyphenol markers (isosakuranetin, norwogonin‐glucuronide, pinocembrin, enterolactone, and hippuric acid) whose relative levels were associated with lower risk of selected childhood outcomes in the LiNA cohort and provided a exploratory evidence consistent with IL‐5‐related immunomodulation in AD. Nonetheless, several limitations must be acknowledged: semiquantification of metabolites in a single urine sample, a relatively small subcohort with cytokine quantification, potential parental misreporting, lack of microbiome data, and generalizability limited to Central European populations. Our findings are hypothesis‐generating and require replication in larger cohorts with quantitative metabolomics analysis of repeated exposure assessment. This study underscores a gap in approaches that integrate prenatal maternal diet, gut microbiome function, and immune programming to strengthen causal inference.

## Author Contributions

S.G.‐O.: Conceptualization, Formal analysis, Investigation, Methodology, Visualization, Writing – original draft, Writing – review and editing. C.H.: Investigation, Validation. S.R.: Data curation, Methodology. M.B.: Investigation, Resources. M.K.: Funding acquisition, Project administration. W.B.: Resources. A.C.Z.: Funding acquisition, Project administration, Writing – review and editing. G.H.: Conceptualization, Supervision, Writing – original draft, Writing – review and editing. All authors critically revised the manuscript and approved the final version for publication.

## Funding

This work was supported by the European Commission, Number. 101136566.

## Conflicts of Interest

The authors declare no conflicts of interest.

## Supporting information


**Figure S1:** (A) Flow chart of LiNA mothers and paired 3‐year‐old children included in the analysis with data on health outcomes (*n* = 478) and blood immune markers (*n* = 268). (B) Directed acyclic graph that displays the potential causal pathway linking gestational polyphenol marker levels (light blue node) to atopic and respiratory outcomes at age 3 (red node), while controlling for covariates (gray nodes).
**Figure S2:** (A) Box plot presents the distribution of 14 polyphenol markers measured in urine during pregnancy. The compounds are ordered from left to right by detection rate (highest to lowest) within each polyphenol group, as denoted by box color. Note that peak intensities were used as semiquantitative indicators of exposure but are not directly comparable across polyphenol markers. (B) Pairwise Spearman's rank correlation matrix of food marker levels. In the color spectrum, blue and red shades show positive and negative correlations between the compounds. The asterisk (*) denotes statistically significant correlations (*p* < 0.05).
**Figure S3:** Quantile g‐computation model on the association of a mixture of microbiota‐derived polyphenols detected in pregnancy with wheezing and bronchitis in 3‐year‐old children. Models were fitted using polyphenol markers quantized into tertiles (q = 3) and adjusted for smoking/ETS exposure during pregnancy, breastfeeding up to 6 months, cat keeping, parental atopy history, parental education level, and child sex. The plots display the relative contribution (weights) of each compound to the overall mixture estimate, with bars indicating the direction (positive or negative) of the partial associations. Darker bar shading denotes a stronger overall association with the outcome. The overall estimates (adjusted OR and 95% CI) and *p*‐values for each outcome are shown in the table.
**Table S1:** Distribution of blood immune marker concentrations, including fx5‐IgE [kU/L] and type 2 cytokines [pg/mL], quantified in children aged 3 years (*n* = 268).
**Table S2:** Description of sociodemographic characteristics and outcomes of mother–child pairs in the entire LiNA cohort with polyphenol measurements at pregnancy (*n* = 581/622) and in the 3‐year follow‐up (*n* = 478), and the subcohort with polyphenol data and IgE/cytokine quantification (*n* = 268) in children aged 3 years.
**Table S3:** Characteristics, sources, and detection rates of food markers measured in the LiNA cohort (*n* = 581).

## Data Availability

The data that support the findings of this study are available on request from the corresponding author. The data are not publicly available due to privacy or ethical restrictions.
