# Supplementary material for: Gestational Polyphenol Levels and Risk of Atopic and Respiratory Outcomes in Early‐Life: Insights From the LiNA Study
Source: Allergy. 2026 Apr 3;81(7):2544–8. doi: 10.1111/all.70328 (PMC13342753; doi:10.1111/all.70328)
Supplement: Supplementary file 1 — Figure S1: (A) Flow chart of LiNA mothers and paired 3‐year‐old children included in the analysis with data on health outcomes (n = 478) and blood immune markers (n = 268). (B) Directed acyclic graph that displays the potential causal pathway linking gestational polyphenol marker levels (light blue node) to atopic and respiratory outcomes at age 3 (red node), while controlling for covariates (gray nodes). Figure S2: (A) Box plot presents the distribution of 14 polyphenol markers measured in urine during pregnancy. The compounds are ordered from left to right by detection rate (highest to lowest) within each polyphenol group, as denoted by box color. Note that peak intensities were used as semiquantitative indicators of exposure but are not directly comparable across polyphenol markers. (B) Pairwise Spearman's rank correlation matrix of food marker levels. In the color spectrum, blue and red shades show positive and negative correlations between the compounds. The asterisk (*) denotes statistically significant correlations (p < 0.05). Figure S3: Quantile g‐computation model on the association of a mixture of microbiota‐derived polyphenols detected in pregnancy with wheezing and bronchitis in 3‐year‐old children. Models were fitted using polyphenol markers quantized into tertiles (q = 3) and adjusted for smoking/ETS exposure during pregnancy, breastfeeding up to 6 months, cat keeping, parental atopy history, parental education level, and child sex. The plots display the relative contribution (weights) of each compound to the overall mixture estimate, with bars indicating the direction (positive or negative) of the partial associations. Darker bar shading denotes a stronger overall association with the outcome. The overall estimates (adjusted OR and 95% CI) and p‐values for each outcome are shown in the table. Table S1: Distribution of blood immune marker concentrations, including fx5‐IgE [kU/L] and type 2 cytokines [pg/mL], quantified in children aged 3 years (n = 268 [file ALL-81-2544-s001.docx]

**Supporting Information for**

**Gestational Polyphenol Levels and Risk of Atopic and Respiratory Outcomes in Early-Life: Insights from The LiNA Study**

Gómez-Olarte et al.

**Detailed Method Description**

**Description of the LiNA study**

The study was conducted with data from the prospective birth cohort LiNA: Lifestyle and environmental factors and their influence on the Newborn Allergy risk, involving 629 mother-child pairs, of which 622 correspond to mothers and 629 to children (7 twin pairs)[^1^](#_ENREF_1). The pregnant women were recruited between May 2006 and December 2008 in Leipzig, Germany. All participants voluntarily provided written informed consent. The LiNA study was approved by the Institutional Review Board of the University of Leipzig (file ref. No. 046-2006). Mothers and children were monitored by annual follow-ups, with biological sample collection (blood and urine) and detailed self-reported standardized questionnaires. For the present study, cytokine and IgE were quantified in the children's blood samples at age 3 years, and polyphenol markers were detected in urine samples from the third trimester of pregnancy as previously described[^2^](#_ENREF_2). Lifetime prevalence of children’s health outcomes, including atopic dermatitis (AD), wheezing, and bronchitis, and the relevant covariates were defined using longitudinal ISAAC-based questionnaire data from pregnancy to the 3-year follow-up. To control for potential confounding, known environmental and lifestyle variables, including smoking/environmental tobacco smoke (ETS) exposure during pregnancy, breastfeeding up to 6 months, cat keeping, parental atopy history, parental education level, and child sex, were included as covariates in the statistical models (directed acyclic graph, **Figure S1B**).

**Quantification of food allergen-specific IgE and type 2 cytokines**

IgE specific for food allergens (fx5) was quantified in the serum of children aged 3 years using the Phadia ImmunoCAP System (Thermo Fisher Scientific, Freiburg, Germany). The allergen sources tested in the fx5 panel comprised hen’s egg, cow’s milk, wheat, fish, peanut, and soy. Food sensitization was defined based on the fx5 allergen-specific IgE serum concentration >0.35 kU/L[^3^](#_ENREF_3). For cytokine measurements, heparinized blood from children aged 3 years was prepared within 6 h of drawing. Whole blood samples (500 μL) were incubated for 4 h at 37°C with the mitogen phytohemagglutinin (PHA, 50 μg/mL; Sigma Aldrich, Hamburg, Germany). Thereafter, the samples were diluted 1:1 with RPMI 1640 medium without supplements and then centrifuged. Collected cell-free supernatants were stored at -80°C until analysis. Concentrations of the type 2 cytokines IL-4, IL-5, IL-10, and IL-13 were quantified using a cytometric bead array (BD CBA Human Soluble Flex Set system; Becton Dickinson, Heidelberg, Germany), according to the manufacturer’s instructions (**Table S1**).

**Measurement of polyphenol markers**

The suspect and non-targeted screening for polyphenols is described in Huber et al., 2024[^2^](#_ENREF_2). In brief, first-morning void urine samples from 581 women in the 34^th^-36^th^ week of pregnancy were analyzed by liquid chromatography coupled to high-resolution mass spectrometry (LC-HRMS). After the extraction of signal intensities from ion chromatograms for the whole sample set, 46 polyphenol compounds were identified through tandem spectral library search (Schymanski level 2a)[^4^](#_ENREF_4). Annotated polyphenols with detection rates (DRs) >70% (14 out of 46) were selected for further regression analyses (**Table S3**/**Figure S2A**).

**Statistical analysis**

For hypothesis testing, *p*-values < 0.05 were considered statistically significant, and those < 0.10 were deemed borderline significant. Missing data on the covariates smoking (n = 2) and breastfeeding (n = 18) were completed using multiple imputation (m = 20) by chained equations with logistic regression models. Given the semiquantitative nature of the food marker measurements, polyphenol values were categorized into tertiles (T), setting T1 as the reference. The collinearity across the exposure matrix was examined based on Spearman’s rank correlation coefficients, as polyphenols often derive from common food sources (e.g., vegetables and fruits). Crude and adjusted multivariable logistic regression models were used to assess the associations between 14 polyphenol markers measured in pregnancy and children’s health outcomes at age 3. To account for multiple testing across the food metabolites, *p*-values were adjusted using the Benjamini-Hochberg false discovery rate (FDR) procedure. Moreover, quantile g-computation models were applied to investigate potential associations with mixtures of flavonoids, the larger polyphenol subgroup (n = 9) in the exposure set, and microbiota-derived metabolites (n = 3, **Table S3**). Health outcomes exhibiting borderline or statistically significant associations with at least one polyphenol marker within these subgroups were selected for mixture analyses, which were adjusted for the covariates defined a priori. For the mixture framework, continuous polyphenol markers were internally quantified into tertiles (q = 3) and modeled as ordinal scores (0-2). Thus, the mixture estimates from quantile g-computation analyses denote the association of each health outcome with a one-tertile increase across all exposures. This method integrates individual logistic regression coefficients (β) into an overall estimate of the association between the mixture (correlated exposure matrix) and outcome. The combined positive and negative scaled estimates are computed by summing up each β coefficient with matching signs, which indicate the direction of the association with the outcome. To rank each exposure contribution (weight) to the mixture model, β coefficients are divided by the overall estimate (positive or negative)[^5^](#_ENREF_5). Mixture estimates and 95% CI were calculated by the bootstrapping method (300 iterations) and presented as ORs to improve interpretability.

Mediation analyses were conducted to examine whether children’s type 2 cytokine concentrations (IL-4, IL-5, IL-10, and IL-13) were consistent with the associations between polyphenol markers and AD at age 3. To maximize contrast, the analysis was constrained to mother-child pairs ranked in the lowest and upper exposure tertiles (T3 vs. T1, n = 178). The outcome and mediator models were fitted using AD as a binary response variable and log_2_-transformed type 2 cytokine concentrations as continuous variables. Independent mediation models were used to estimate the direct and indirect associations, named average causal mediation effect (ACME) in this framework, between selected polyphenol markers and AD via each type 2 cytokine. Based on these estimates, the average direct effect (ADE) that captures the mediator-independent association with the outcome was calculated (**Figure 2B**). The total effect, which represents the combined association of both pathways, and the proportion mediated were also reported. A non-parametric bootstrap procedure (1,000 simulations) was applied to compute estimates (logORs).

Statistical analyses, multiple imputations, and plots were generated using the R software (v4.4.1).

**Supporting Tables**

**Table S1.** Distribution of blood immune marker concentrations, including fx5-IgE [kU/L] and type 2 cytokines [pg/mL], quantified in children aged 3 years (n = 268).

| **Protein** | **Units** | **Median** | **Min** | **Percentile** | | **Max** |
| --- | --- | --- | --- | --- | --- | --- |
|  |  |  |  | **25^th^** | **75^th^** |  |
| fx5-IgE | kU/L | 0.07 | 0.01 | 0.04 | 0.16 | 8.74 |
| IL-4 | pg/mL | 23.46 | 1.99 | 8.92 | 59.20 | 425.71 |
| IL-5 |  | 5.42 | 2.05 | 2.53 | 13.32 | 93.69 |
| IL-10 |  | 85.84 | 2.71 | 57.48 | 124.45 | 609.96 |
| IL-13 |  | 41.94 | 2.50 | 20.54 | 69.07 | 312.55 |

Abbreviations. Min: minimum value; Max: maximum value.

**Table S2.** Description of sociodemographic characteristics and outcomes of mother-child pairs in the entire LiNA cohort with polyphenol measurements at pregnancy (n = 581/622) and in the 3-year follow-up (n = 478), and the subcohort with polyphenol data and IgE/cytokine quantification (n = 268) in children aged 3.

| **Characteristic, n (%)** | **Entire cohort ^a^**  **n = 581** | **3-year follow-up ^b^**  **n = 478** | **Subcohort ^c^ n = 268** | ***p*-value ^d^** |
| --- | --- | --- | --- | --- |
| Maternal age at delivery (years) | |  |  | 0.812 |
| < 25 | 61 (10%) | 42 (8.8%) | 23 (8.6%) |  |
| 25-30 | 223 (38%) | 178 (37%) | 109 (41%) |  |
| 30-35 | 197 (34%) | 167 (35%) | 90 (34%) |  |
| > 35 | 100 (17%) | 91 (19%) | 46 (17%) |  |
| Smoking/ETS exposure during pregnancy | |  |  | 0.417 |
| No | 470 (83%) | 407 (86%) | 234 (88%) |  |
| Yes | 98 (17%) | 69 (14%) | 33 (12%) |  |
| Missing | 13 | 2 | 1 |  |
| Breastfeeding (up to 6 months) | |  |  | 0.356 |
| No | 121 (22%) | 94 (20%) | 61 (23%) |  |
| Yes | 417 (78%) | 366 (80%) | 200 (77%) |  |
| Missing | 43 | 18 | 7 |  |
| Cat keeping | |  |  | 0.876 |
| No | 476 (82%) | 392 (82%) | 221 (82%) |  |
| Yes | 105 (18%) | 86 (18%) | 47 (18%) |  |
| Family history of atopy | |  |  | 0.615 |
| None | 192 (33%) | 159 (33%) | 83 (31%) |  |
| One parent | 274 (47%) | 229 (48%) | 127 (47%) |  |
| Both parents | 115 (20%) | 90 (19%) | 58 (22%) |  |
| Parental school education ^e^ | |  |  | 0.728 |
| Low | 16 (2.8%) | 5 (1.0%) | 2 (0.7%) |  |
| Medium | 128 (22%) | 103 (22%) | 64 (24%) |  |
| High | 437 (75%) | 370 (77%) | 202 (75%) |  |
| Child sex | |  |  | 0.851 |
| Male | 303 (52%) | 246 (51%) | 136 (51%) |  |
| Female | 278 (48%) | 232 (49%) | 132 (49%) |  |
| **Outcome prevalence at year 3 ^f^** | | | | |
| Atopic dermatitis |  |  |  | 0.436 |
| No | 440 (86%) | 408 (85%) | 223 (83%) |  |
| Yes | 74 (14%) | 70 (15%) | 45 (17%) |  |
| Wheezing |  |  |  | 0.401 |
| No | 314 (61%) | 295 (62%) | 157 (59%) |  |
| Yes | 200 (39%) | 183 (38%) | 111 (41%) |  |
| Bronchitis |  |  |  | 0.258 |
| No | 258 (50%) | 240 (50%) | 123 (46%) |  |
| Yes | 256 (50%) | 238 (50%) | 145 (54%) |  |

^a^ Mothers of the entire LiNA cohort with complete questionnaire and maternal food marker data at pregnancy/birth, n = 581/622.

^b^ Mother-child pairs of the 3-year follow-up with complete questionnaire data and food marker measurements at pregnancy.

^c^ Subcohort of mother-child pairs of the 3-year follow-up with complete questionnaire data, food marker measurements at pregnancy, and children’s blood protein quantification. ^d^ The Chi-square or Fisher’s exact test (n <5).

^e^ Parental education was defined based on the number of schooling years: ≤9 years (low), 10 years (medium), and ≥12 years (high).

^f^ The first column displays the children of the 3-year follow-up (n = 514) with complete questionnaire data.

**Table S3.**Characteristics, sources, and detection rates of food makers measured in the LiNA cohort (n = 581).

| **Polyphenol marker** | **DR (%)** | **Group** | **Flavonoid subclass** | **Sources** |
| --- | --- | --- | --- | --- |
| Naringenin | 90.0 | Fruit flavonoids | Flavanone | Citrus fruits |
| Cyanidin-glycoside | 79.7 |  | Anthocyanin | Berries, red fruits |
| Homoeriodictyol | 79.0 |  | Flavanone | Citrus fruits |
| Isosakuranetin | 74.3 |  | Flavanone | Citrus fruits |
| Phloretin | 73.8 |  | Dihydrochalcone | Apples, pears |
| Norwogonin-glucuronide | 73.6 |  | Flavone | Herbs |
| Pinocembrin | 77.7 | Plant flavonoids | Flavanone derivative | Honey/propolis |
| Daidzein | 75.6 |  | Isoflavone | Soy-derived phytoestrogen |
| 8-prenylnaringenin | 71.2 |  | Flavanone | Found in hops/beer |
| Enterolactone | 98.6 | Microbiota-derived polyphenols | Lignan metabolite | Gut microbiota-derived lignan metabolite |
| Hippuric acid | 86.8 |  | Polyphenol metabolite | General dietary phenolic metabolite |
| Urolithin | 76.0 |  | Microbiota metabolite | Formed by gut bacteria from ellagitannins |
| Riboflavin | 99.8 | Vitamin | Vitamin B₂ | Mostly dairy, meat, and fortified foods |
| 34-dimethoxycinnamic acid | 95.1 | Plant polyphenol | Phenylpropanoid derivative | Plant-based foods |

Abbreviations. DR: detection rate.

**Supporting Figures**


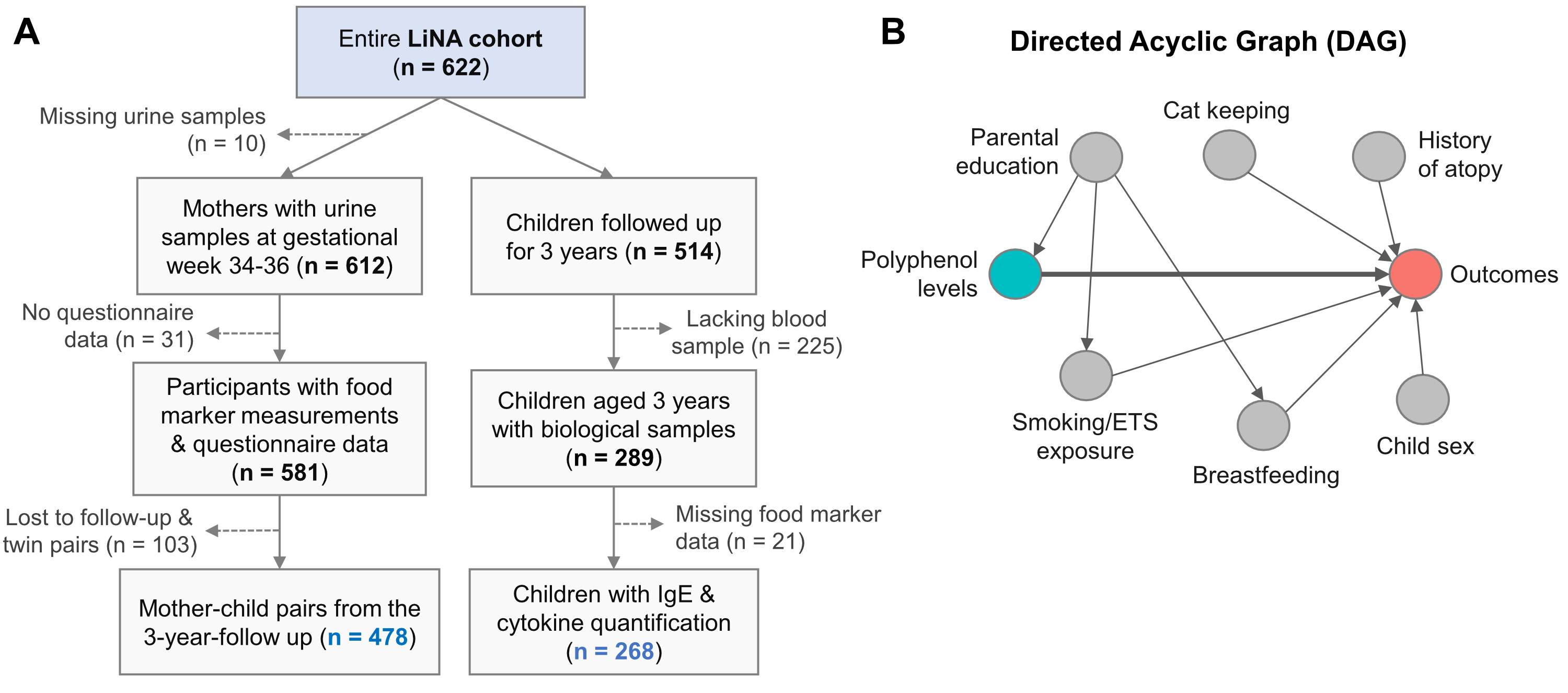


**Figure S1.** (**A**) Flow chart of LiNA mothers and paired 3-year-old children included in the analysis with data on health outcomes (n = 478) and blood immune markers (n = 268). (**B**) Directed acyclic graph that displays the potential causal pathway linking gestational polyphenol marker levels (light blue node) to atopic and respiratory outcomes at age 3 (red node), while controlling for covariates (grey nodes).


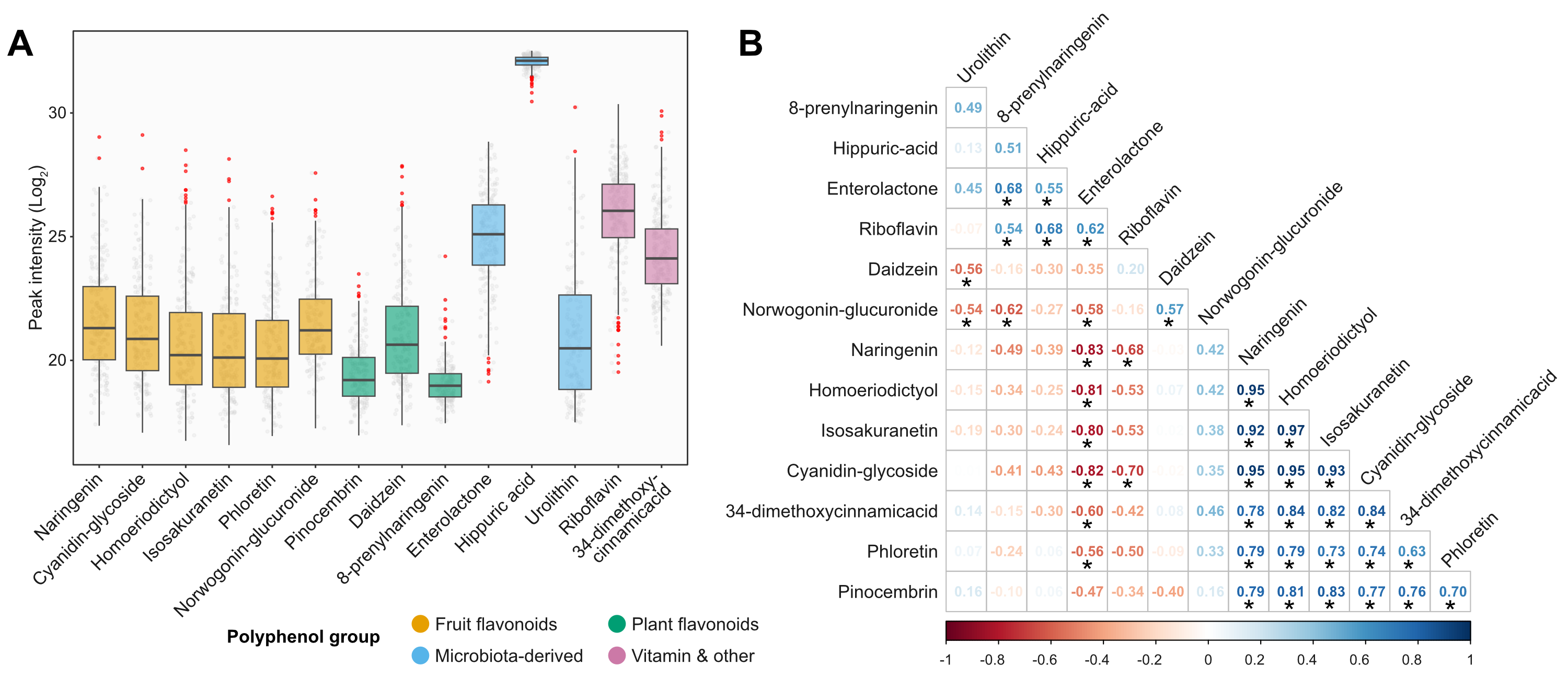


**Figure S2.** (**A**) Box plot that presents the distribution of 14 polyphenol markers measured in urine during pregnancy. The compounds are ordered from left to right by detection rate (highest to lowest) within each polyphenol group, as denoted by box color. Note that peak intensities were used as semiquantitative indicators of exposure, but are not directly comparable across polyphenol markers. (**B**) Pairwise Spearman’s rank correlation matrix of food marker levels. In the color spectrum, blue and red shades show positive and negative correlations between the compounds. The asterisk (*) denotes statistically significant correlations (*p* <0.05).


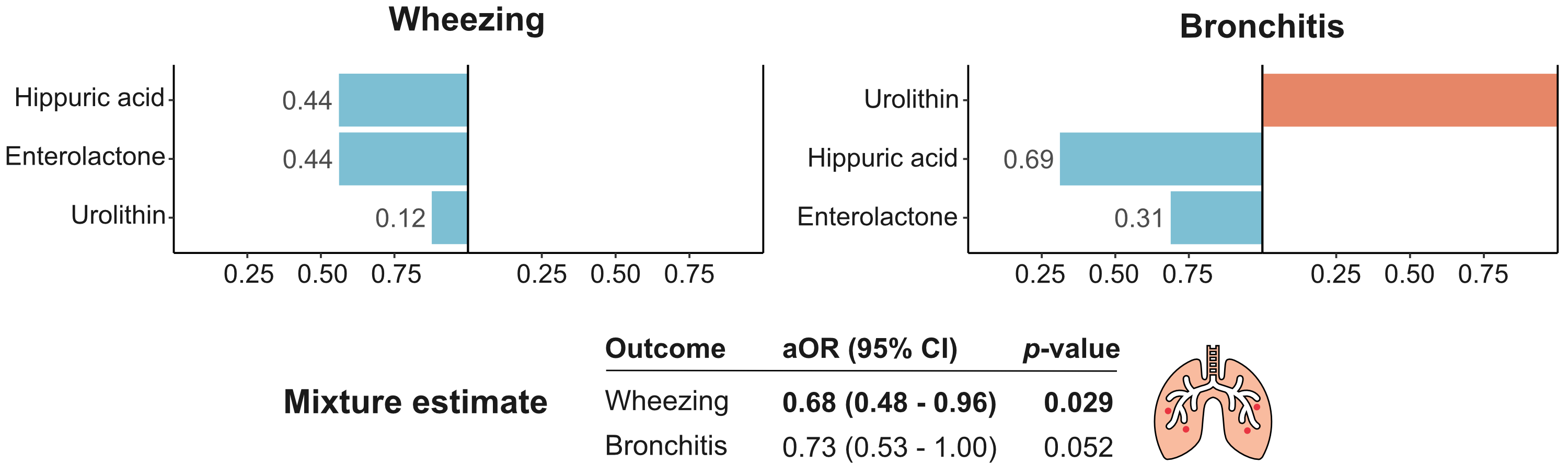


**Figure S3.** Quantile g-computation model on the association of a mixture of microbiota-derived polyphenols detected in pregnancy with wheezing and bronchitis in 3-year-old children. Models were fitted using polyphenol markers quantized into tertiles (q=3) and adjusted for smoking/ETS exposure during pregnancy, breastfeeding up to 6 months, cat keeping, parental atopy history, parental education level, and child sex. The plots display the relative contribution (weights) of each compound to the overall mixture estimate, with bars indicating the direction (positive or negative) of the partial associations. Darker bar shading denotes a stronger overall association with the outcome. The overall estimates (adjusted OR and 95% CI) and *p*-values for each outcome are shown in the table.

**Additional references**

1. Hinz D, Bauer M, Röder S, et al. Cord blood Tregs with stable FOXP3 expression are influenced by prenatal environment and associated with atopic dermatitis at the age of one year. *Allergy.* 2012;67(3):380-389.

2. Huber C, Brack W, Röder S, et al. Pesticide residues and polyphenols in urine - A combined LC-HRMS screening to reveal intake patterns. *Environ Int.* 2024;191:108981.

3. Ballardini N, Nilsson C, Nilsson M, Lilja G. ImmunoCAP Phadiatop Infant--a new blood test for detecting IgE sensitisation in children at 2 years of age. *Allergy.* 2006;61(3):337-343.

4. Schymanski EL, Jeon J, Gulde R, et al. Identifying small molecules via high resolution mass spectrometry: communicating confidence. *Environ Sci Technol.* 2014;48(4):2097-2098.

5. Keil AP, Buckley JP, O'Brien KM, Ferguson KK, Zhao S, White AJ. A Quantile-Based g-Computation Approach to Addressing the Effects of Exposure Mixtures. *Environ Health Perspect.* 2020;128(4):47004.
